# Cross-talk between lipopolysaccharide tolerance and AGEs in the regulation of macrophage inflammation and cholesterol efflux

**DOI:** 10.3389/fimmu.2026.1669028

**Published:** 2026-02-12

**Authors:** Danielle Ribeiro Santos, Monique de Fatima Mello Santana, Eduarda Palanca, Milena Gomes Vancini, Sayonara Ivana Santos de Assis, Aritania Sousa Santos, Denise Frediani Barbeiro, Suely Kunimi Kubo Ariga, Maria Lucia Correa-Giannella, Francisco Garcia Soriano, Marisa Passarelli

**Affiliations:** 1 Laboratório de Lípides (LIM10), Hospital das Clínicas (HCFMUSP) da Faculdade de Medicina da Universidade de Sao Paulo, Sao Paulo, Brazil; 2 Programa de Pós-Graduação em Medicina, Universidade Nove de Julho, Sao Paulo, Brazil; 3 Laboratório de Carboidratos e Radioimunoensaio (LIM18), Hospital das Clínicas (HCFMUSP) da Faculdade de Medicina da Universidade de Sao Paulo, São Paulo, Brazil; 4 Laboratório de Emergências Clínicas (LIM51), Hospital das Clínicas (HCFMUSP) da Faculdade de Medicina da Universidade de Sao Paulo, Sao Paulo, Brazil

**Keywords:** advanced glycation, atherosclerosis, inflammation, lipopolysaccharide tolerance, macrophages

## Abstract

**Introduction:**

Immune cell infiltration with high expression of receptors for advanced glycation end products (RAGE) and Toll-like receptor 4 (TLR4) promotes vascular inflammation and accelerates atherosclerosis. Advanced glycated albumin (AGE-albumin) primes macrophages for heightened inflammatory responses to lipopolysaccharide (LPS). Here, we investigated whether LPS-induced tolerance modulates AGE-driven inflammatory priming and cholesterol efflux in macrophages.

**Methods:**

Cholesterol-enriched bone marrow–derived macrophages (BMDMs) and RAW264.7 macrophages were subjected to LPS tolerance induction, treated with control (C)- or AGE-albumin, and rechallenged with LPS. In parallel, LPS tolerance was induced *in vivo* by repeated low-dose LPS injections, followed by BMDM differentiation, cholesterol loading, albumin treatments, and secondary LPS stimulation. Tumor necrosis factor (TNF) secretion was assessed by ELISA, gene expression by RT-qPCR, and HDL-mediated ¹^4^C-cholesterol efflux using conditioned media or direct HDL incubation.

**Results:**

In BMDMs, LPS tolerance reduced TNF secretion following C-albumin treatment but not AGE-albumin. In RAW264.7 macrophages, TNF secretion was reduced by 53% and 77.6% after C- and AGE-albumin treatment, respectively. BMDMs from LPS-tolerant mice exhibited reduced TNF secretion following both albumin treatments. Gene expression analysis revealed that AGE-albumin selectively increased *Ager* and *Tlr4* expression in tolerant BMDMs, whereas C-albumin was associated with broad suppression of pro-inflammatory genes. Conditioned media from tolerant BMDMs markedly enhanced HDL-mediated cholesterol efflux in naïve macrophages, while direct exposure of tolerant BMDMs to AGE-albumin reduced HDL-mediated efflux by 40%.

**Discussion:**

These findings demonstrate that LPS tolerance promotes an atheroprotective macrophage phenotype characterized by attenuated inflammatory signaling and enhanced cholesterol efflux. However, this protective immunometabolic program is selectively disrupted by AGE exposure, highlighting a critical interface through which chronic metabolic stress may override innate immune tolerance and contribute to atherosclerotic progression.

## Introduction

Atherosclerosis development is accompanied by the progressive establishment of inflammatory processes that ultimately contribute to apoptotic and thrombogenic events (1). Cardiovascular (CV) risk factors such as dyslipidemia, hypertension, smoking, obesity, diabetes mellitus (DM), and infectious processes promote inflammatory insults to the arterial wall. Indeed, elevated levels of inflammatory cytokines and opsonins are predictive of acute coronary events and prospectively define CV risk (2). The athero-inflammatory profile is characterized by the early infiltration of monocytes into the arterial intima, followed by phenotypic polarization of macrophages toward the pro-inflammatory M1 subtype, along with the presence of T and B lymphocytes, mast cells, dendritic cells, neutrophils, eosinophils, and basophils (3), all of which secrete inflammatory cytokines. Monocytes/macrophages are the principal cells involved in pathogen recognition and, alongside other immune cells, exhibit robust phagocytic capacity, supported by high expression of scavenger receptors, receptors for advanced glycation end-products (RAGE), toll-like receptors (TLRs), and others that mediate rapid clearance of pathogens and modified macromolecules from the microenvironment. However, chronic activation of these receptors initiates intracellular pathways associated with oxidative and inflammatory stress, contributing to the progression of atherosclerosis.

The role of inflammation in atherogenesis is further supported by the observation that treatment with pharmacological agents, such as statins, which lower cholesterol levels and oxidative events, reduces inflammation and helps prevent CV disease (CVD) (4, 5). Moreover, pharmacological inhibition of interleukin-1 beta has been shown to decrease CV risk independently of plasma lipid levels (6).

Advanced glycation end products (AGEs) are common in diabetes mellitus (DM) and chronic kidney disease, and they can also come from external sources like diet, tobacco, and pollution. The glycation process occurs through a non-enzymatic reaction between glucose or oxoaldehydes and proteins, lipids, and nucleic acids, leading to the irreversible formation of AGEs. These AGEs interact with RAGE, triggering nuclear factor kappa B (NFKB)-mediated inflammatory genes, and they also alter protein structure and function, causing changes in the extracellular matrix that promote vascular damage. The signaling pathways of the AGE-RAGE and lipopolysaccharide (LPS)-TLR4 axes converge and enhance the cellular inflammatory response. These receptors share several ligands, including AGEs, LPS, high mobility group box 1, and calgranulins. The adaptor proteins myeloid differentiation factor 88 (MyD88) and toll/interleukin-1 receptor (TIR) domain-containing adaptor protein (TIRAP) are mediators in both TLR4 and RAGE signaling pathways; the latter involves binding to the cytoplasmic domain of RAGE after phosphorylation by protein kinase C (PKC) (7–9). This canonical pathway recruits interleukin-1 receptor-associated kinases (IRAK) and tumor necrosis factor receptor-associated factor 6 (TRAF6), which then recruit receptor-interacting protein 1 (RIP-1) and activate the transforming growth factor beta-activated kinase 1 (TAK1) complex. This complex activates mitogen-activated protein kinases (MAPKs), ultimately leading to the activation of activator protein 1 (AP-1), a transcription factor that moves to the nucleus and regulates inflammatory gene expression (10). Finally, NFKB is activated, enhancing the expression of inflammation-related genes through the coordinated and amplified signaling of RAGE and TLR4 (8, 9).

Cellular responses to LPS through TLRs trigger the production of reactive oxygen species and nitric oxide. The magnitude of this response is influenced by both the LPS dose and the frequency of exposure, giving rise to the phenomenon known as LPS tolerance. This adaptive state is characterized by reduced cellular responsiveness to subsequent LPS challenges, including several mechanisms that regulate the intensity and duration of TLR-induced inflammation, such as the inhibition of TLR signaling by negative regulatory molecules, the production of anti-inflammatory cytokines, and modifications in downstream post-receptor signaling cascades. Together, these processes lead to TLR desensitization and the establishment of LPS-induced hypo-responsiveness, a hallmark of endotoxin tolerance (11).

Previous studies have shown that AGEs prime macrophages for a sustained inflammatory response upon LPS stimulation, thereby impairing high-density lipoprotein (HDL)-mediated cholesterol efflux (12, 13). This mechanism likely sensitizes the arterial wall microenvironment to inflammation and lipid accumulation, contributing to atherosclerosis by downregulating ATP-binding cassette transporter A1 (ABCA1) in macrophages (12). In the present study, it was investigated how LPS tolerance, both *in vitro* and *in vivo*, modulates inflammation elicited by AGEs and its impact on HDL-mediated cholesterol efflux.

## Material and methods

### Lipoproteins isolation

The study was reviewed and approved by the Research Ethics Committee of the Clinical Hospital of the Faculty of Medicine, University of São Paulo (Hospital das Clínicas da Faculdade de Medicina da Universidade de São Paulo – CAPPesq #2.397.639). Written informed consent to participate in this study was provided by the participants. Blood samples were collected from healthy individuals following a 12-hour fasting period. Plasma was immediately isolated by centrifugation at 4 °C for 15 minutes at 3000 rpm (Thermo Scientific™ Sorvall™ ST centrifuge, Osterode am Harz, Germany) following the addition of preservatives (µL/mL of plasma): 20 µL of 0.25% gentamicin/chloramphenicol, 5 µL of 0.5% aprotinin, 5 µL of 2 mM benzamidine, and 0.5 µL of phenylmethylsulfonyl fluoride (PMSF) diluted in 30 mM dimethyl sulfoxide (Sigma-Aldrich, Steinheim, Germany). Low-density lipoprotein (LDL; D = 1.019–1.063 g/mL) and HDL (D = 1.063–1.21 g/mL) were isolated by discontinuous density gradient ultracentrifugation. Lipoprotein fractions were dialyzed against phosphate-buffered saline (PBS; NaCl 137 mmol/L; Na_2_HPO_4_ 4 mmol/L; KCl 2 mmol/L; K_2_HPO_4_ 1 mmol/L) containing 0.4 g/L EDTA (pH 7.4) for 24 hours at 4 °C, followed by sterilization using 0.22 μm filters (Millex^®^, Merck KGaA, Darmstadt, Germany). Protein concentration in the samples was determined using the Lowry method (14).

### LDL acetylation

LDL was acetylated according to the protocol described by Basu et al. (1976) (15), following extensive dialysis against PBS and sterilization.

### Modification of albumin by advanced glycation

Fatty acid-free bovine serum albumin (Sigma-Aldrich) was incubated in the presence of a freshly prepared 10 mM glycolaldehyde solution (Sigma-Aldrich, Fluka-Buchs, Germany) in PBS containing 0.4 g/L EDTA (pH 7.4) to induce the formation of AGE-albumin. Control albumin (C-albumin) was incubated in PBS containing 0.4 g/L EDTA only. All incubations were carried out under sterile conditions, in a nitrogen atmosphere, at 37 °C in a water bath with gentle agitation for 4 days. Samples were then dialyzed against PBS and sterilized using 0.22 μm filters. Endotoxin levels in the albumin samples were determined using the Limulus Amebocyte Lysate (LAL) assay (Cape Cod, Falmouth, MA, USA), and only samples with endotoxin concentrations below 50 pg/mL were used in the experiments. The final protein concentration was determined, and samples were stored at –70 °C until use. Total AGEs and pentosidine contents in C-albumin and AGE-albumin were assessed by fluorescence. Briefly, one hundred microliters of albumin samples were mixed with 100 µL of PBS containing EDTA in black 96-well microplates (Costar, Black). Fluorescence measurements were performed using a Synergy HT Multi-Mode Microplate Reader (BioTek^®^, Winooski, VT, USA). Samples were excited at 370 nm, and emission was measured at 440 nm and 378 nm for total AGEs and pentosidine, respectively.

### Bone marrow-derived macrophages

Macrophages were differentiated from bone marrow-derived cells isolated from C57BL/6 and BALB/c mice, aged 6 to 12 weeks. Animals were euthanized by intraperitoneal injection of an overdose of ketamine hydrochloride (300 mg/kg body weight) and xylazine hydrochloride (30 mg/kg body weight), following the guidelines of the Brazilian National Council for the Control of Animal Experimentation (CONCEA) under the Ministry of Science, Technology, and Innovation (MCTI). The experimental protocol was approved by the Animal Ethics Committee (CEUA) of the Faculdade de Medicina da Universidade de São Paulo (#1912/2023). Femurs and tibias from both hind limbs were dissected to obtain undifferentiated cells. The epiphyses of the bones were removed, and bone marrow was flushed using Dulbecco’s Modified Eagle Medium (DMEM) supplemented with antibiotics, fetal calf serum (FCS), and conditioned medium derived from L929 cells. Bone marrow was gently aspirated to dissociate cellular aggregates. Cells were centrifuged (1000 rpm, 6 min, room temperature) and seeded in culture plates, followed by incubation in a CO_2_ incubator for 5 days. On day five, the medium was replaced with fresh conditioned medium to support macrophage growth and differentiation. On day six, cells were incubated in low-glucose DMEM supplemented with 10% FCS. After reaching confluence, bone marrow-derived macrophages (BMDMs) were used for the assessment of inflammatory activity (cytokine secretion) and cholesterol efflux assays.

### RAW 264.7 macrophages

RAW 264.7 macrophage cell line was cultured in 75 cm² flasks using Roswell Park Memorial Institute (RPMI 1640) medium supplemented with 10% fetal bovine serum. For experimental procedures, cells were detached by gentle scraping, seeded into 24-well plates (4 × 10^5^ cells/well), and incubated for 24 hours in serum-free RPMI medium to allow adhesion.

### LPS tolerance in macrophages

RAW 264.7 cells and BMDMs were loaded with acetylated LDL (50 µg/mL) for 24 hours and subsequently challenged or not with lipopolysaccharide (LPS, 1 µg/mL) for an additional 24 hours to induce LPS tolerance. LPS-tolerant and non-tolerant cells were then treated with either C-albumin or AGE-albumin (2 mg/mL) for 48 hours, followed by a second LPS stimulation (5 µg/mL) for 24 hours.

### Cell viability

Cell viability was assessed following the experimental procedures using Trypan Blue exclusion and a lactate dehydrogenase (LDH) assay (Thermo Fisher Scientific, Wilmington, DE, USA).

### LPS tolerance induction in mice

Male BALB/c mice, 7 weeks old (n = 20), were kept under a 12-hour light/dark cycle with free access to standard chow and water. The LPS-tolerant group (n = 10) received a daily subcutaneous injection of LPS (1 mg/kg; *Escherichia coli* serotype 026: B6, Sigma) for five consecutive days. Control animals (n = 10) received no intervention. Two days after the last LPS injection, the animals were anesthetized, and bone marrow cells were harvested and differentiated into macrophages as previously described. The BMDMs were then treated with acetylated LDL, C-albumin, or AGE-albumin, followed by LPS challenge. Cholesterol efflux, gene expression, and inflammatory profiles were then assessed.

### Inflammatory cytokines measurement

Concentration of the pro-inflammatory cytokine TNF in the culture supernatant was measured by ELISA (R&D Systems, USA), as previously described (Okuda et al., 2012).

### Gene expression

The expression of genes related to the AGE/RAGE/TLR4 axis and inflammatory response was assessed in BMDMs. Total RNA was extracted using the TRIzol^®^ reagent (Gibco BRL, Life Technologies Research Products, Grand Island, NY, USA), following the manufacturer’s instructions. RNA integrity was evaluated with a NanoDrop spectrophotometer (ND1000, Thermo Fisher Scientific). Complementary DNA (cDNA) was synthesized from 500 ng of total RNA using the High-Capacity RNA-to-cDNA Kit (Applied Biosystems, Foster City, CA, USA) and a Mastercycler thermal cycler (Eppendorf, Hamburg, Germany). Gene expression analysis was conducted using FAM-labeled probes from the TaqMan^®^ Gene Expression Assays (Applied Biosystems) for the following genes: *Tlr4* (Mm00445273_m1), *Ager* (Mm00545815_m1), *Nfkb1* (Mm00476361_m1), *Rela* (Mm00501346_m1), *Il6* (Mm00446190_m1), *Tnf* (Mm00443258_m1) and *Abca1* (Mm004426_m1). Real-time quantitative PCR reactions were performed in triplicate on a StepOnePlus™ Real-Time PCR System (Applied Biosystems), and data were analyzed with StepOne Software version 2.1 (Applied Biosystems) (13). Relative gene expression was normalized to the housekeeping gene *Actb* (Mm02619580_g1) and calculated using the comparative cycle threshold method (2^−ΔΔCt) (16).

### Determination of HDL-mediated ¹^4^C-cholesterol efflux

#### Macrophages treated with conditioned medium

Naïve BMDMs were loaded with acetylated LDL (50 µg/mL) and ¹^4^C-cholesterol (0.3 µCi/mL; GE Healthcare, Bucks, UK), for 48 hours. After thorough washing, cells were incubated for 24 hours with a pooled sample of conditioned culture medium from the previously described experimental conditions. Cells were then washed with PBS supplemented with fatty acid-free albumin (FAFA) and incubated with HDL (50 µg/mL) for 6 hours.

#### Macrophages of tolerant or non-tolerant mice

LPS tolerance was induced in mice as previously described. Bone marrow cells were obtained from LPS-tolerant and non-tolerant mice and differentiated under standard conditions. After differentiation, BMDMs were loaded with acetylated LDL (50 µg/mL) and ¹^4^C-cholesterol (0.3 µCi/mL; GE Healthcare, Bucks, UK), for 24 hours, followed by treatment with either AGE-albumin or C-albumin (2 mg/mL) for 48 hours. To functionally confirm the establishment of LPS tolerance, BMDMs were subsequently challenged with LPS (5 µg/mL) for 24 hours. Cells were incubated with HDL (50 µg/mL) for 6 hours to assess cholesterol efflux. Between each treatment step, cells were thoroughly washed with PBS supplemented with fatty acid–free albumin to remove residual stimuli before the subsequent incubation. The culture medium was collected into glass tubes, centrifuged at 1500 rpm for 10 minutes at 4 °C to remove cell debris, and transferred to scintillation vials. Scintillation fluid (Perkin Elmer, Turku, Finland) was added for radioactivity measurement. Wells were washed twice with cold physiological saline (4 °C), and cellular lipids were extracted using a hexane: isopropanol solution (3:2; Merck, Darmstadt, Germany). After solvent evaporation, cellular radioactivity was measured. Specific HDL-mediated cholesterol efflux was calculated by subtracting basal efflux (measured in the presence of DMEM/FAFA alone). The percentage of cholesterol efflux was determined as: (¹^4^C-cholesterol in the medium/[¹^4^C-cholesterol in the medium + ¹^4^C-cholesterol in the cells]) × 100 (13).

### Statistical analysis

Statistical analysis was conducted using the GraphPad Prism software (version 5.04) for Windows and Microsoft^®^ Excel for Mac (version 16.52). The normality of the samples was assessed using the Shapiro–Wilk test, and group comparisons were performed using the Mann-Whitney test. A value of p < 0.05 was considered statistically significant.

## Results

**Figure 1:**
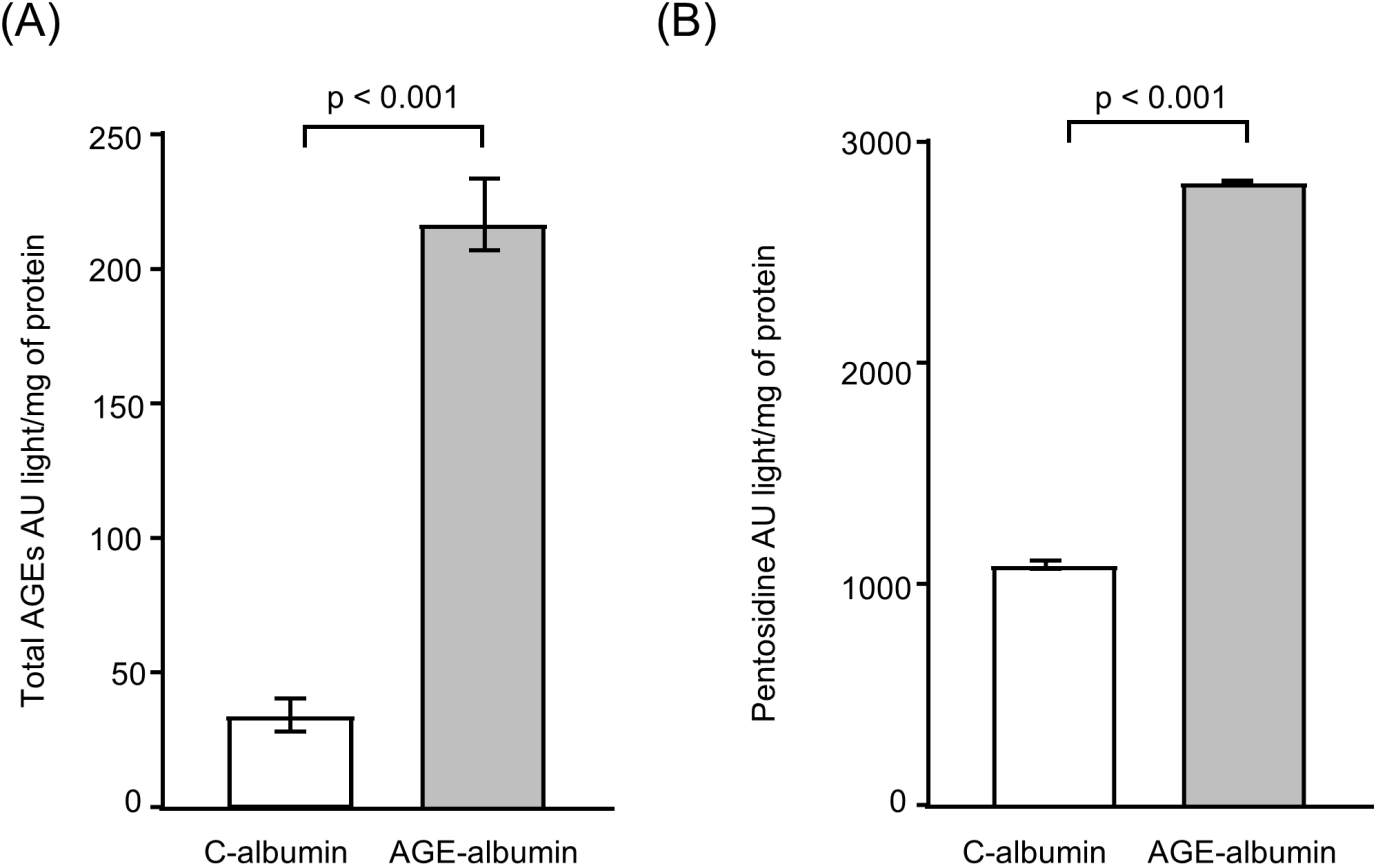
Total AGEs and pentosidine content in C-albumin and AGE-albumin. **(A)** Total AGEs levels measured by fluorimetry (excitation at 370 nm and emission at 440 nm). **(B)** Pentosidine levels measured by fluorimetry (excitation at 370 nm and emission at 378 nm). The amount of total AGEs and pentosidine in C-albumin and AGE-albumin was determined by fluorimetry. Comparisons were performed using the Mann–Whitney test (n = 3).

The total AGEs and pentosidine contents were determined by fluorimetry and were found to be, respectively, 6.5-fold and 2.5-fold higher in AGE-albumin compared to C-albumin (**Figure 1**). Cell viability was verified by trypan blue exclusion, with values consistently above 95% before the experiments. Additionally, the LDH assay confirmed that toxicity was similar in all conditions (data not shown).

**Figure 2:**
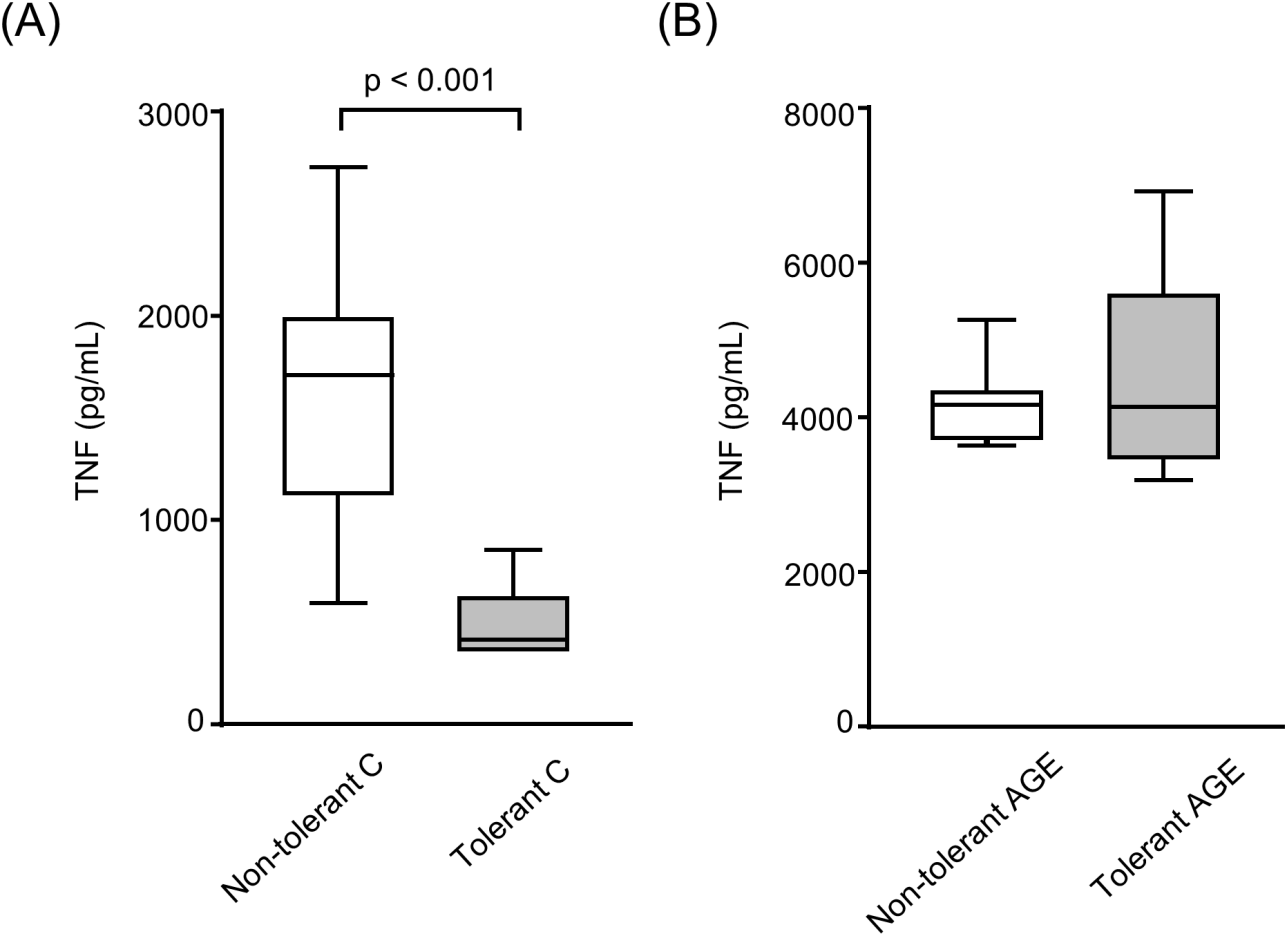
Secretion of tumor necrosis factor (TNF) by LPS-tolerant and nontolerant BMDMs treated with C- or AGE-albumin. BMDMs were loaded with acetylated LDL (50 µg/mL) for 24 hours and subsequently challenged or not with LPS (1 µg/mL) for an additional 24 hours. LPS-tolerant and non-tolerant cells were then treated with either control albumin (C-albumin) or AGE-albumin (2 mg/mL) for 48 hours, followed by a second LPS stimulation (5 µg/mL) for 24 hours. TNF concentration in the culture supernatant was measured by ELISA. **(A)** Non-tolerant Calbumin vs tolerant C-albumin. **(B)** Non-tolerant AGE-albumin vs tolerant AGEalbumin. Comparisons were performed using the Mann–Whitney test. Data are presented as medians with interquartile ranges (25th–75th percentiles; n = 8). The figure is representative of two independent experiments.

In LPS-tolerant BMDMs, TNF secretion was reduced by 45% following treatment with C-albumin and subsequent LPS challenge (**Figure 2A**). However, treatment with AGE-albumin was able to override this established tolerance, as no reduction in TNF secretion was observed after the final LPS stimulation (**Figure 2B**).

**Figure 3:**
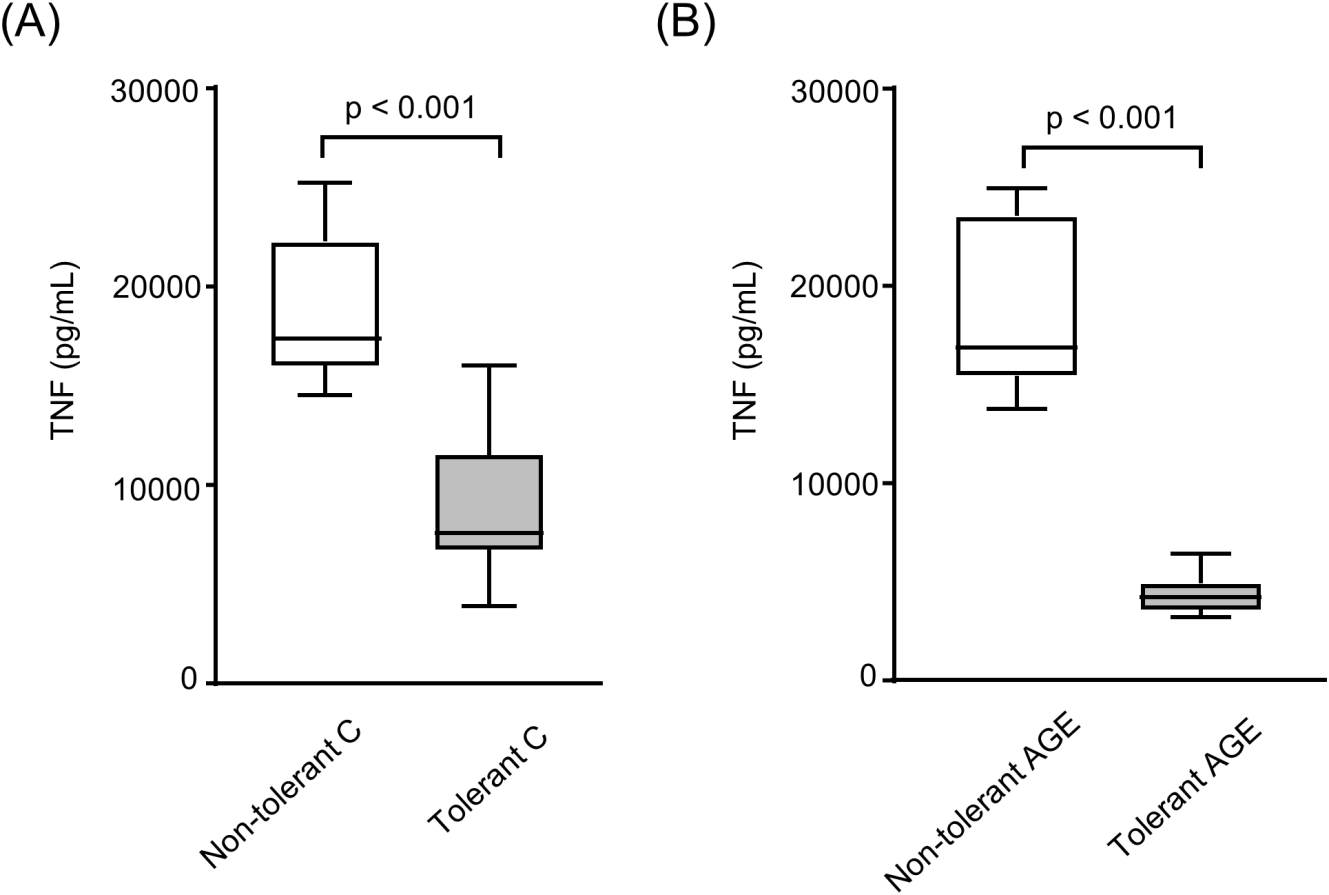
Secretion of TNF by LPS-tolerant and non-tolerant RAW 264.7 macrophages treated with control or AGE-albumin. RAW 264.7 macrophages were loaded with acetylated LDL (50 µg/mL) for 24 hours and subsequently challenged or not with LPS (1 µg/mL) for an additional 24 hours. LPS-tolerant and non-tolerant cells were then treated with either C-albumin or AGE-albumin (2 mg/mL) for 48 hours, followed by a second LPS stimulation (5 µg/mL) for 24 hours. TNF concentration in the culture supernatant was measured by ELISA. **(A)** Non-tolerant C-albumin vs tolerant Calbumin. **(B)** Non-tolerant AGE-albumin vs tolerant AGE-albumin. Comparisons were performed using the Mann–Whitney test. Data are presented as medians with interquartile ranges (25th–75th percentiles; n = 8). The figure is representative of two independent experiments.

In contrast, LPS-tolerant RAW264.7 macrophages exhibited a 77.6% reduction in TNF secretion following treatment with AGE-albumin, compared to non-tolerant cells (**Figure 3B**). Similarly, tolerance reduced TNF secretion by 53% in cells treated with C-albumin (**Figure 3A**). Thus, unlike the findings in BMDMs, prior LPS exposure in RAW macrophages effectively induced tolerance that was sufficient to prevent AGE-induced inflammatory sensitization upon subsequent LPS challenge (**Figure 3**).

**Figure 4:**
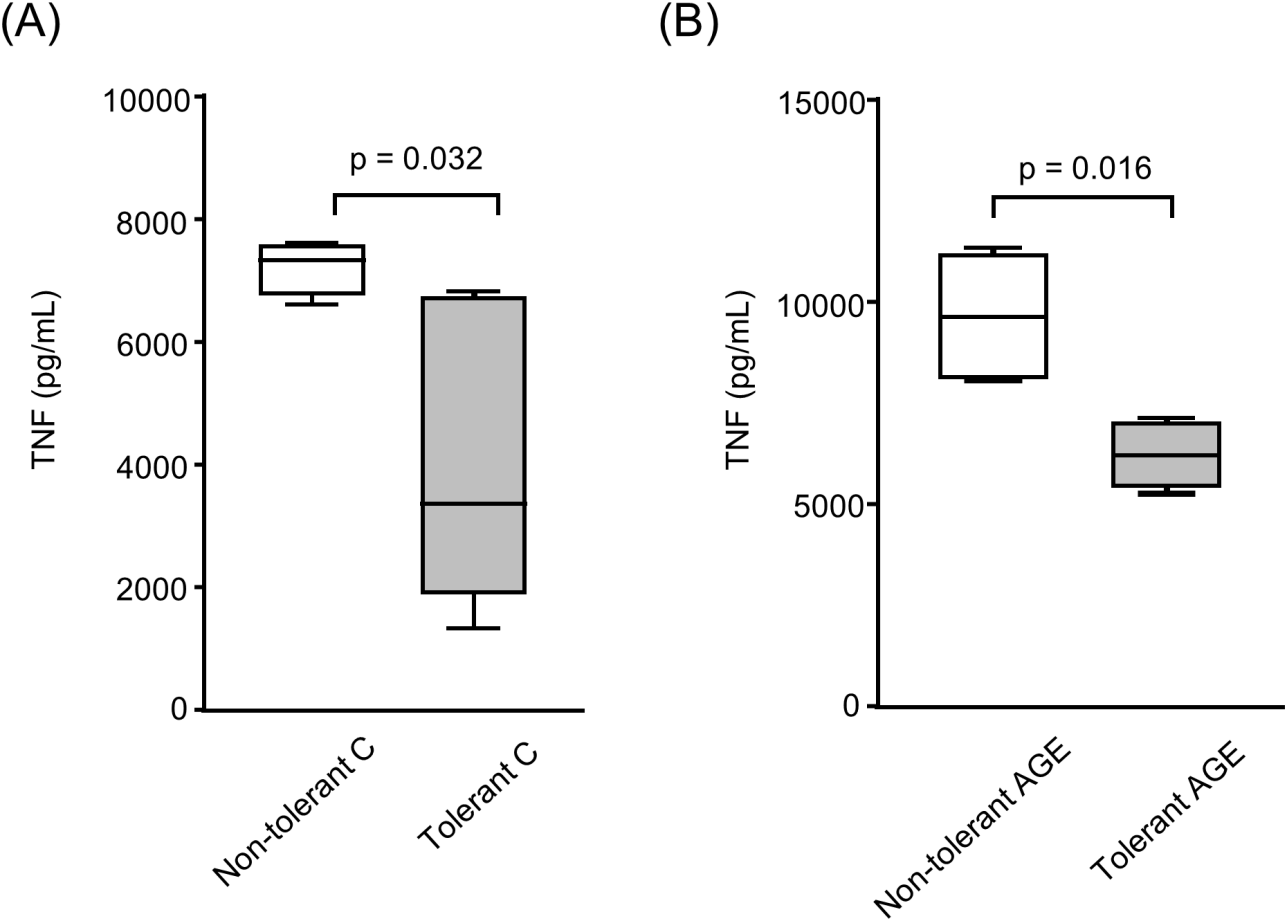
Secretion of TNF by BMDMs derived from LPS-tolerant and nontolerant mice. BMDMs were differentiated from BALB/c mice two days after subcutaneous administration of LPS (1 mg/kg) for five consecutive days to induce tolerance, or from untreated control (non-tolerant) animals. Cells were loaded with acetylated LDL (50 µg/mL) for 24 hours, followed by treatment with either C-albumin or AGE-albumin (2 mg/mL) for 48 hours. Subsequently, cells were challenged with LPS (5 µg/mL) for 24 hours. TNF concentration in the culture supernatant was quantified by ELISA. **(A)** Non-tolerant C-albumin vs tolerant C-albumin. **(B)** Non-tolerant AGEalbumin vs tolerant AGE-albumin. Comparisons were performed using the Mann–Whitney test. Data are presented as medians with interquartile ranges (25th–75th percentiles; n = 8). The figure is representative of two independent experiments.

To investigate the systemic impact of prior LPS exposure, an *in vivo* model was employed. Mice were rendered tolerant through repeated LPS administration. Following this, TNF secretion was reduced by 35% in BMDMs obtained from tolerant mice treated with AGE-albumin, suggesting that LPS tolerance attenuated inflammatory mediator release even in the presence of AGE stimulation (**Figure 4B**).

**Figure 5:**
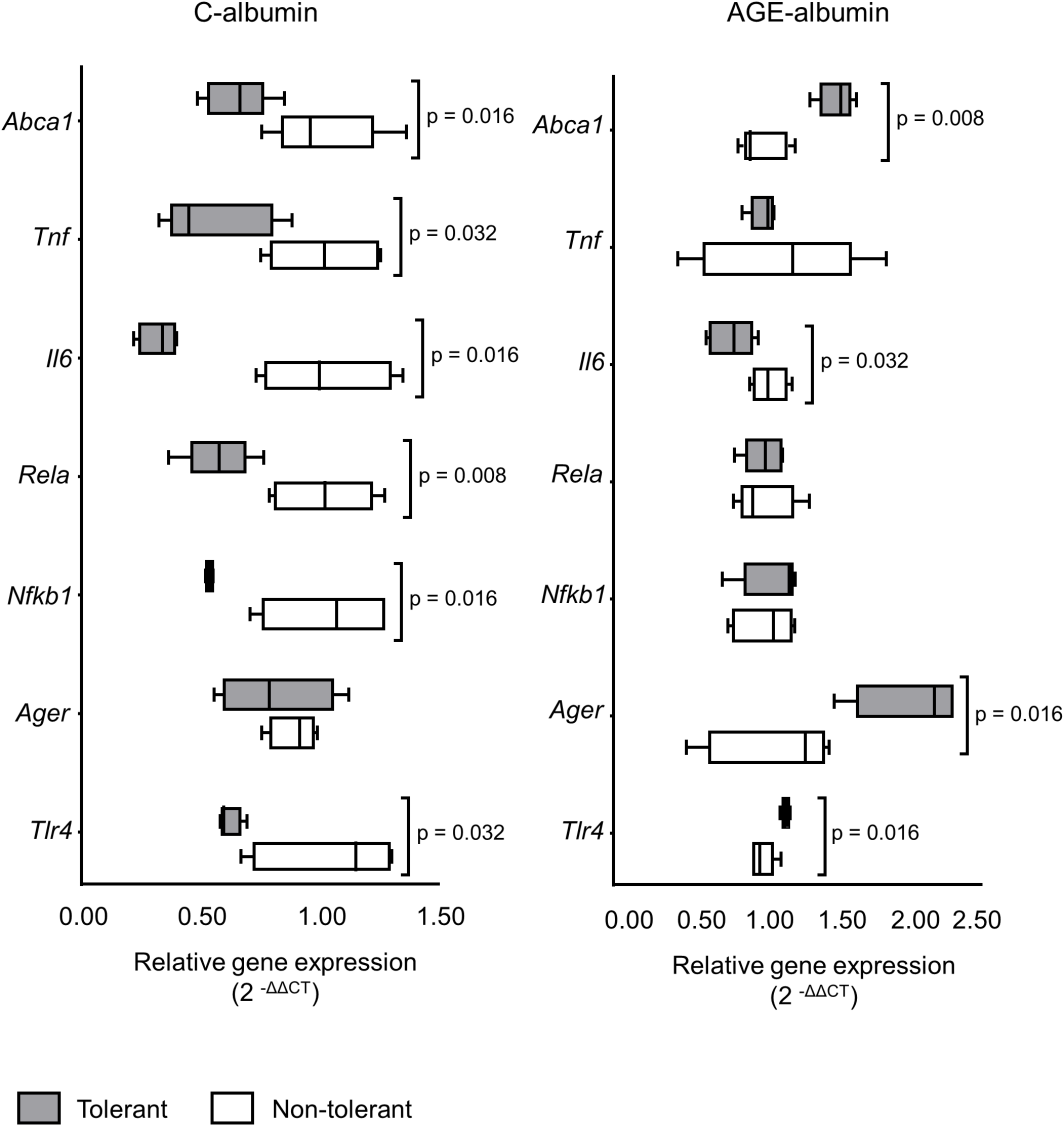
Expression of genes involved in the AGE-RAGE-TLR axis and inflammation in BMDMs derived from LPS-tolerant and non-tolerant Mice. BMDMs were differentiated from BALB/c mice two days after subcutaneous administration of LPS (1 mg/kg) for five consecutive days to induce tolerance, or from untreated control (non-tolerant) animals. Cells were loaded with acetylated LDL (50 µg/mL) for 24 hours, followed by treatment with either C-albumin or AGE-albumin (2 mg/mL) for 48 hours. Subsequently, cells were challenged with LPS (5 µg/mL) for 24 hours. RT-qPCR was performed, and relative gene expression was normalized to the *Actb* housekeeping gene. The quantification of relative expression levels was carried out using the comparative cycle threshold method (2^−ΔΔCt) (n=5). Comparisons were performed using the Mann–Whitney test.

Alterations in gene expression associated with the AGE signaling pathway and inflammatory response were also observed. *Tlr4* expression was reduced by 40% in BMDMs from LPS-tolerant animals treated with C-albumin but increased by 16% in cells treated with AGE-albumin (**Figure 5**). In LPS-tolerant mice, AGE-albumin treatment increased *Ager* expression by 59% compared to non-tolerant controls (**Figure 5**).

Expression of *Nfkb1* and *Rela*, encoding the p50 and p65 subunits of NFKB, respectively, was reduced by 48% and 43% in macrophages from LPS-tolerant mice treated with C-albumin (**Figure 5**). This expression pattern is consistent with reduced basal NFKB activation in LPS tolerance. However, no significant changes in the expression of these genes were observed in AGE-albumin-treated cells (**Figure 5**).

*Tnf* and *Il6* expression were markedly elevated in non-tolerant animals treated with AGE-albumin and subsequently challenged with LPS, approximately 15-fold and 10^4^-fold increases, respectively (**Figure 5**). In contrast, *Il6* expression was reduced by 68% in C-albumin-treated tolerant cells and by 25% in AGE-albumin-treated tolerant cells (**Figure 5**).

Expression of *Abca1* was 36% lower in C-albumin-treated tolerant animals compared to non-tolerant controls, while AGE-albumin treatment increased *Abca1* expression by 50% in tolerant cells relative to non-tolerant ones (**Figure 5**).

**Figure 6:**
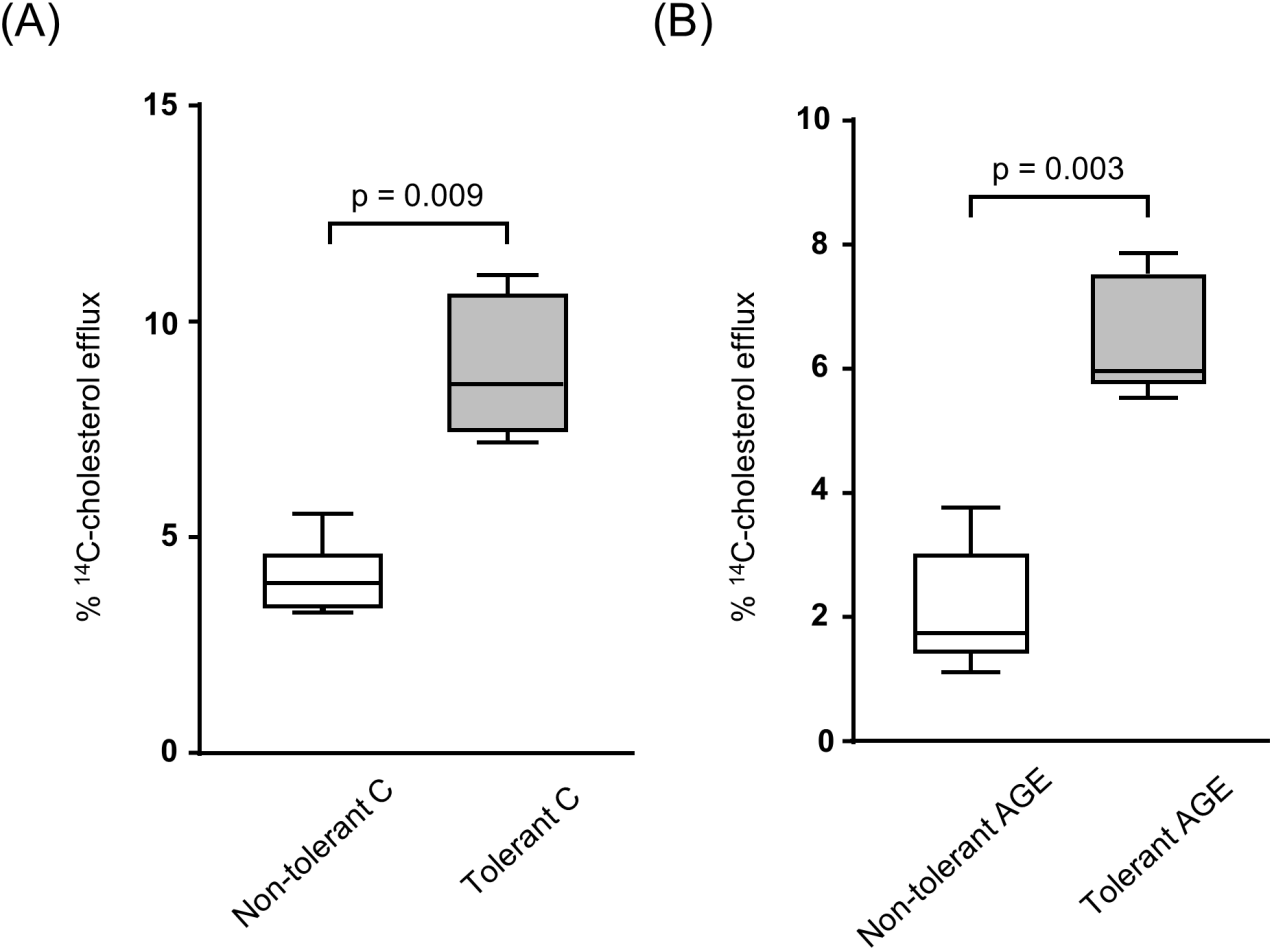
HDL-mediated ^14^C-cholesterol efflux in naïve BMDMs treated with conditioned media derived from tolerant and non-tolerant BMDMs. Naïve BMDMs were loaded with acetylated LDL (50 µg/mL) and ^14^C-cholesterol (0.3 µCi/mL) for 48 hours and subsequently treated for 24 hours with conditioned media obtained from BMDMs derived from LPS-tolerant or non-tolerant mice, previously treated with Calbumin or AGE-albumin and challenged with LPS. To assess cholesterol efflux, cells were incubated in the presence or absence of HDL (50 µg/mL) for 6 hours. **(A)** Conditioned medium from non-tolerant vs tolerant BMDMs treated with C-albumin. **(B)** Conditioned medium from non-tolerant vs tolerant BMDMs treated with AGE-albumin. Comparisons were performed using the Mann–Whitney test. Data are presented as medians with interquartile ranges (25th–75th percentiles; n = 8). The figure is representative of two independent experiments.

To assess cholesterol efflux, naïve BMDMs were incubated with conditioned media from macrophages of tolerant and non-tolerant mice. Cells treated with media from tolerant animals showed increased cholesterol efflux: a twofold increase with C-albumin (**Figure 6A**) and a threefold increase with AGE-albumin (**Figure 6B**). These results suggest that LPS tolerance *in vivo* may reduce cytokine secretion and influence lipid homeostasis in macrophages.

**Figure 7:**
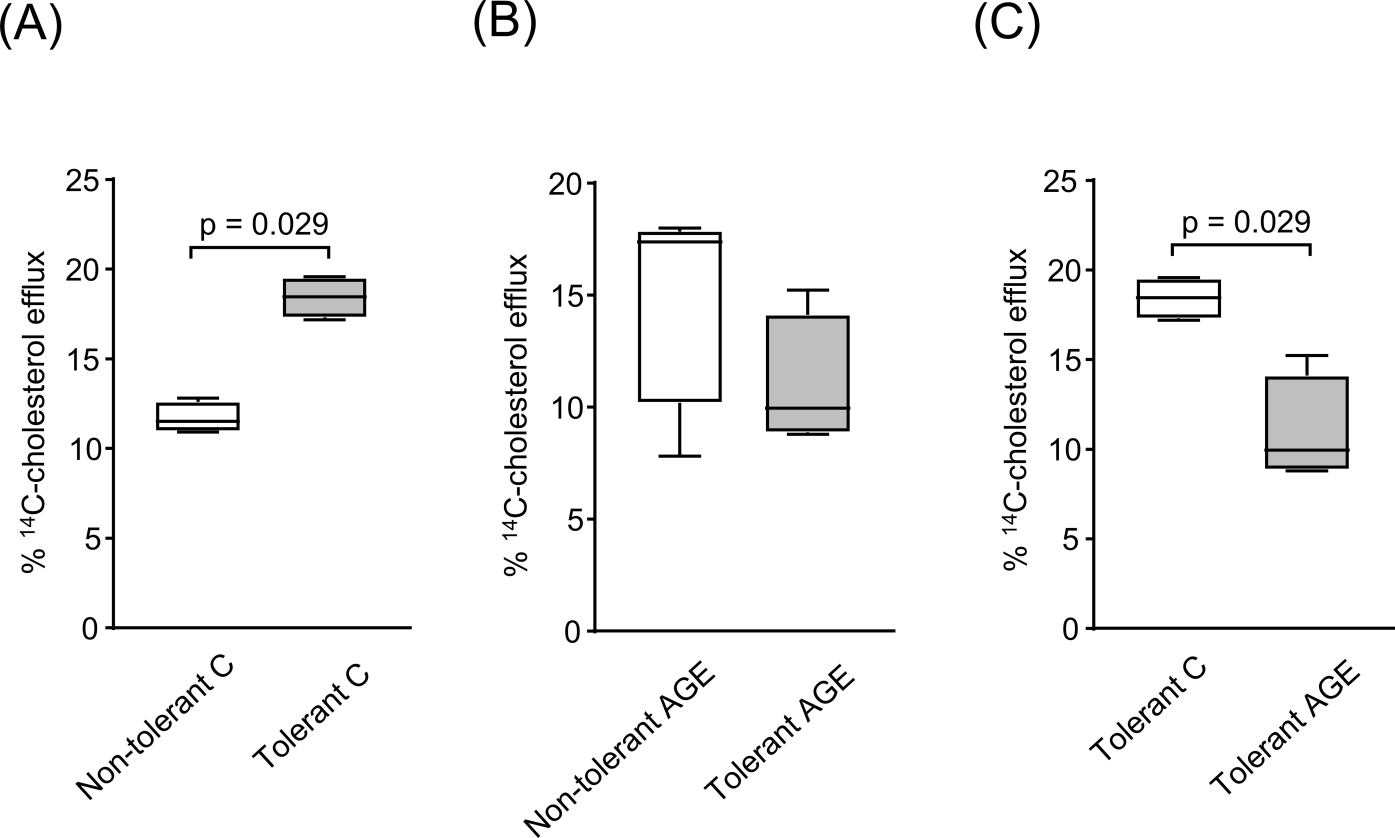
HDL-mediated ^14^C-cholesterol efflux in BMDMs derived from tolerant and non-tolerant mice. BMDMs obtained from LPS-tolerant or non-tolerant mice were loaded with acetylated LDL (50 µg/mL) and ^14^C-cholesterol (0.3 µCi/mL) for 24 hours, followed by treatment with either C-albumin or AGE-albumin for 48 hours. Cells were then challenged with LPS, after which cholesterol efflux was assessed by incubation in the presence or absence of HDL (50 µg/mL) for 6 hours. **(A)** Non-tolerant vs tolerant BMDMs treated with C-albumin. **(B)** Non-tolerant vs tolerant BMDMs treated with AGE-albumin. **(C)** Tolerant BMDMs treated with C-albumin or AGE-albumin. Statistical comparisons were performed using the Mann–Whitney test. Data are presented as medians with interquartile ranges (25th–75th percentiles; n = 4).

To determine whether LPS tolerance exerts a direct *in vivo* effect independent of conditioned media, BMDMs were isolated from LPS-tolerant or non-tolerant mice and exposed to conditions mimicking an atherosclerotic milieu, following treatment with C-albumin or AGE-albumin. LPS tolerance markedly increased HDL-mediated cholesterol efflux by 57% in BMDMs treated with C-albumin (**Figure 7A**). In contrast, no tolerance-associated enhancement of HDL-mediated cholesterol efflux was observed in BMDMs exposed to AGE-albumin (**Figure 7B**). Furthermore, in LPS-tolerant BMDMs, AGE-albumin treatment reduced cholesterol efflux by approximately 40% compared with C-albumin (**Figure 7C**). Collectively, these results demonstrate that LPS tolerance promotes a protective HDL-dependent cholesterol efflux response, which is selectively abrogated by the presence of AGEs.

## Discussion

In the present study, the effects of AGE-albumin on inflammatory insult in macrophages were evaluated, focusing on the signaling interface between AGEs and LPS. Both converge through RAGE and TLR4 to activate inflammatory pathways that can be modulated by LPS tolerance.

AGEs are independently associated with the development of CVD, largely by altering lipid metabolism, impairing reverse cholesterol transport, and exacerbating inflammatory insult (17–20). Moreover, activation of the AGE-RAGE axis has been shown to upregulate matrix metalloproteinases (MMPs) (21). In particular, AGEs increase MMP-9 expression via the RAGE-MAPK-NFKB signaling pathway (21–23), thereby contributing to plaque vulnerability by promoting thinning of the fibrous cap and increasing the risk of plaque rupture (24).

Inflammation underlies the progression of atherosclerosis and other chronic diseases. Alterations in lipid homeostasis, typically characterized by intracellular accumulation of cholesterol and toxic oxysterols, trigger inflammatory processes in cells infiltrated in the arterial wall, thereby aggravating atherosclerotic lesions (25).

In BMDMs, LPS tolerance induced by prior *in vitro* treatment with LPS effectively reduced TNF secretion following incubation with C-albumin and a subsequent LPS challenge. However, no difference in TNF secretion was observed between tolerant and non-tolerant cells incubated with AGE-albumin. These results indicate that AGE-albumin was able to override LPS tolerance, consistent with previous studies showing that AGE-albumin sensitizes macrophages to inflammatory stimulation by LPS, rendering them more reactive, with increased transcription of inflammatory genes and elevated secretion of TNF, IL-6, and VCAM-1 (12). The effects of AGEs were sustained even after their removal from the culture medium for up to 24 hours (13), a phenomenon attributed to persistent expression of the *Rela* gene and nuclear activation of the p65 subunit of NFKB in macrophages (26). NFKB is a central mediator in the activation of inflammatory genes, primarily through the nuclear heterodimerization of its p50 and p65 subunits. Interestingly, p65 expression is elevated in endothelial cells following transient hyperglycemia and remains upregulated for six days, even after the cells were returned to normoglycemic conditions (27). Epigenetic modifications, including enhanced histone methylation (H3K4me1) in the proximal promoter region of the gene encoding the p65 subunit, have been observed in mice experiencing transient hyperglycemia (27).

Since BMDMs are considered naïve due to the lack of prior challenge, experiments were also conducted with RAW264.7 macrophages. In these cells, LPS tolerance reduced TNF secretion by 52.9% and 77.6% in macrophages treated with C-albumin and AGE-albumin, respectively. Notably, in RAW macrophages, TNF secretion following a second LPS stimulus was even lower in cells treated with AGE-albumin compared to those treated with C-albumin, both groups having undergone prior *in vitro* LPS stimulation. These findings suggest that tolerance may be a cell-type-specific phenomenon capable of mitigating inflammatory responses, warranting further detailed investigation into the underlying molecular mechanisms.

The potential existence of cross-tolerance between AGEs and LPS should be considered. In human monocytes, low doses of AGE-albumin induce tolerance rather than sensitization, as evidenced by reduced induction of inflammatory cytokines upon a second LPS challenge compared to cells without prior treatment with AGE (28).

AGEs induce inflammatory stress via multiple pathways, which may also explain their potential to counteract LPS tolerance. These mechanisms include activation of endoplasmic reticulum (ER) stress, reflected by increased expression of chaperones and proteins involved in the unfolded protein response (29). Chronic ER stress is linked to inflammatory and apoptotic pathways that trigger atherosclerotic plaque rupture (30). The activation of the ER-associated degradation (ERAD) is linked to the ubiquitin-proteasome system (31), which degrades ABCA1 (32, 33). This process amplifies inflammatory insults by promoting the intracellular accumulation of sterols and impairing the anti-inflammatory functions attributed to ABCA1 (34, 35). Furthermore, AGEs have been implicated in activation of the NLRP3 inflammasome, leading to pyroptotic cell death (36). The balance between exacerbation and resolution of inflammation is likely critical in the progression of atherosclerotic lesions and the development of acute coronary syndrome.

LPS or endotoxin tolerance involves exposing cells to low doses of LPS for a defined period, which induces a state of hyporesponsiveness to the endotoxin, characterized by reduced secretion of inflammatory cytokines compared to cells without prior exposure (37, 38). The underlying mechanisms of tolerance primarily include, in a synergistic manner, altered expression of pattern recognition receptors (PRRs), epigenetic, and metabolic reprogramming. Upon initial LPS stimulation, macrophages upregulate PRRs and recognize pathogen-associated molecular patterns (PAMPs), such as LPS (39). This leads to an altered secondary response characterized by modulated secretion of inflammatory cytokines, including TNF and IL-6 (40). Studies in human monocytes have demonstrated that treatment with high doses of LPS induces repressive histone modifications (H3K9me2 and H3K27me2), resulting in decreased expression of pro-inflammatory cytokines and a suppressed secondary response (41, 42).

BMDMs prolonged exposed to LPS have increased expression of microRNA-221 (miR-221) and miR-222, which regulate the *Brg1* gene encoding Brahma-related gene 1 protein, a key factor in chromatin remodeling. This event is followed by transcriptional silencing of specific inflammatory genes and signal transducers and activators of transcription protein (STAT)-mediated chromatin remodeling, culminating in immunological tolerance to subsequent challenges. Collectively, these findings highlight the regulatory role of miRs in the functional reprogramming of macrophage memory (43). Additionally, histone deacetylases 1 and 6 decrease histone acetylation at gene transcription regions, thereby establishing immunological tolerance memory in macrophages (44). Interestingly, beyond macrophage reprogramming through LPS tolerance, regulatory T cells (Tregs) have been shown to modulate endotoxin tolerance *in vivo* (45–47). These findings suggest a coordinated crosstalk between innate and adaptive immune pathways that underlies complex mechanisms of inflammatory sensitization and tolerance at the lesion site.

To examine systemic effects, LPS tolerance was induced in mice through consecutive low-dose LPS injections. BMDMs from these tolerant animals showed changes in genes related to the RAGE-TLR4 axis. The expression of *Tlr4* was decreased in BMDMs from tolerant animals treated with C-albumin, reflecting a typical LPS tolerance profile. This aligns with previous studies indicating that prior LPS exposure induces tolerance through multiple mechanisms that suppress TLR4 signaling, including the downregulation of *Tlr4* expression and the activity of negative regulators like suppressor of cytokine signaling 1 (SOCS-1), IRAK-M, and miRs, especially miR-146a. In macrophages and monocytes, miR-146a targets IRAK1 and TRAF6, forming a negative feedback loop that inhibits NFKB-dependent transcription of pro-inflammatory genes (48). It also influences heterochromatin formation dependent on RelB and silences the TNF promoter (49). Conversely, in tolerant cells treated with AGE-albumin, *Tlr4* expression was increased, suggesting that AGEs may disrupt the maintenance of tolerance. This reduction in tolerance could be due to the ability of AGEs to promote a pro-inflammatory state that enhances *Tlr4* transcription, potentially through epigenetic modifications or activation of compensatory pathways. Novakovic et al. (2016) show that certain stimuli can partially reverse tolerance states through chromatin remodeling (50).

The increased expression of *Ager* in macrophages exposed to AGEs is commonly observed, especially in contexts of chronic inflammation mediated by RAGE (51). In the present study, a 59% increase in *Ager* expression was observed in macrophages from LPS-tolerant mice treated with AGE-albumin, compared to cells from non-tolerant animals. This finding indicates that, even in the LPS-induced tolerant state, the presence of AGEs activates the RAGE pathway, suggesting a compensatory role of this route in maintaining inflammatory signaling. This supports the hypothesis that classical LPS tolerance may be bypassed via the AGE-RAGE axis. Furthermore, genes encoding key components of the inflammatory cascade, such as *Nfkb1* and *Rela*, showed reduced expression in macrophages from tolerant animals treated with C-albumin. The *Nfkb1* gene encodes the p50 subunit of the NFKB transcription factor, while *Rela* encodes the p65 subunit. Together, these subunits form the p50/p65 heterodimer, which, upon activation, translocates to the nucleus to promote transcription of pro-inflammatory genes, such as *Tnf* and *Il6* (52). The reduced expression of these genes suggests decreased basal activation of the NFKB pathway, consistent with an LPS tolerance profile. However, after treatment with AGE-albumin-treated, no significant changes in *Nfkb1* and *Rela* expression were observed in macrophages from tolerant animals, similarly to what was seen with *Ager*. Given the reported long-lasting effects of AGEs on macrophages, this inflammatory memory may contribute to the persistence of chronic inflammation, potentially overriding or counteracting the regulatory effects associated with LPS tolerance.

The kinetics of *Tlr* gene expression highly depend on the duration of stimulation. In macrophages, LPS exposure causes an early increase in *Tlr4* expression within the first few hours (usually 2–6 hours), amplifying the inflammatory response. However, *Tlr4* expression significantly decreases after 12–24 hours, especially due to regulatory mechanisms or tolerance states (53, 54). This reduction is driven by negative feedback regulators that suppress pro-inflammatory gene expression after initial activation, helping prevent excessive inflammation and tissue damage (11, 55). Therefore, the results seen at 24 hours post-stimulation in this study should be understood within this time-based regulation of gene expression, which influences both the strength and the type of immune response.

Regarding cholesterol homeostasis, *Abca1* gene expression was significantly decreased by AGEs in macrophages from non-tolerant mice, a relevant pattern since chronic inflammatory stimuli such as AGEs impair cholesterol efflux via ABCA1 (56, 57). Conversely, LPS tolerance increased *Abca1* expression (by 50%) in AGE-treated BMDM from tolerant mice compared to non-tolerant animals under the same conditions. This finding aligns with data from Thompson et al. (2010), which show that LPS tolerance increases ABCA1 expression, facilitating cholesterol and LPS efflux from macrophages (58).

In the present study, conditioned media derived from BMDMs of LPS-tolerant mice enhanced cholesterol efflux in naïve recipient BMDMs, irrespective of whether cells were treated with C-albumin or AGE-albumin, with a more pronounced effect observed in AGE-albumin-treated cells. These findings indicate that the induction of LPS tolerance promotes a macrophage phenotype capable of favoring cholesterol efflux through paracrine mechanisms, even in the presence of pro-inflammatory and sensitizing stimuli such as AGEs.

The use of conditioned media from BMDMs is supported by the pivotal role of paracrine signaling in shaping the arterial intimal microenvironment. Activated macrophages release soluble mediators that modulate the behavior of neighboring immune and vascular smooth muscle cells, thereby inducing functional changes critical for the regulation of inflammation, lipid homeostasis, and ultimately the progression or regression of atherosclerosis (59).

In contrast, when cholesterol-loaded BMDMs from LPS-tolerant and non-tolerant mice were directly exposed to AGE-albumin, AGE treatment markedly reduced the HDL-mediated cholesterol efflux that was otherwise enhanced by *in vivo* LPS tolerance in the presence of C-albumin. Specifically, although BMDMs from tolerant mice exhibited a significant increase in HDL-dependent cholesterol efflux following incubation with C-albumin, this protective effect was abolished upon exposure to AGE-albumin. These observations suggest that AGEs selectively interfere with HDL-dependent cholesterol efflux mechanisms, rather than fully suppressing the tolerant macrophage phenotype.

Specifically, LPS tolerance enhanced *Abca1* gene expression in a manner that supports atheroprotection and modulates the macrophage secretome to foster a microenvironment conducive to maintaining lipid homeostasis within the arterial wall. Notably, BMDMs from LPS-tolerant mice retained this tolerant phenotype even after differentiation from bone marrow, indicating that LPS tolerance induces systemic, long-lasting adaptive changes that persist through the process of cellular maturation (60). Future studies employing *Ager* or *Tlr4* knockout animals, as well as chemical inhibition and/or silencing of RAGE, TLR4, and downstream effectors of the AGE-RAGE/LPS-TLR4 pathways, may help to elucidate the mechanisms of tolerance and sensitization that modulate lipid metabolism. Although changes in *Abca1* gene expression are not consistently reported following AGE exposure, previous studies have demonstrated AGE-induced ABCA1 degradation (32). In contrast, the upregulation of *Abca1* expression observed in the present study likely reflects a more complex regulatory environment, driven by the combined action of multiple soluble factors present in the conditioned medium, and consistent with an established state of immune tolerance, such as that described for LPS-induced tolerance (58, 61).

Alterations in *Abca1* gene expression were not accompanied by measurements of protein content, which may be considered a limitation of the study, given that *Abca1* mRNA levels do not necessarily reflect the final protein abundance due to post-transcriptional regulation. Moreover, it was not determined whether LPS tolerance can prevent the proteasomal degradation of ABCA-1 induced by AGEs (32). Nevertheless, functional assays of cholesterol efflux provided indirect evidence that LPS tolerance favored cellular cholesterol removal.

The assessment of TNF secretion alone as an indicator of tolerance or sensitization in macrophages, although providing novel evidence of an interface between immunological memory and lipid homeostasis in these cells, represents a limitation of this study. Cytokine responses are inherently dynamic and time-dependent, and the evaluation of a single time point with a restricted gene set does not capture the full complexity of these responses. Moreover, an increase in IL-10 is not invariably observed in macrophages or in humans subjected to LPS tolerance, which may reflect a concomitant reduction in pro-inflammatory cytokines (62, 63). Future analyses would benefit from the assessment of a broader panel of pro- and anti-inflammatory cytokines at multiple time points, in association with the modulation of genes involved in RAGE–TLR4 signaling and cholesterol efflux.

The divergent results observed between BMDMs and RAW264.7 cells are intriguing, as biological variability is expected across distinct cell lineages, particularly when comparing immortalized cell lines with naïve primary cells. Nevertheless, these differences do not detract from the principal findings of the present study, as the mechanisms identified are consistent with previously reported data and are interpreted within the appropriate biological context. The use of both cell models further strengthens the translational relevance of the study by providing complementary insights into inflammatory and metabolic responses.

Inflammatory reprogramming in LPS tolerance may exert beneficial effects on CVD, helping to attenuate systemic inflammation associated with vascular dysfunction and atherosclerotic plaque development (64). Thus, understanding the molecular mechanisms regulating AGE action in the context of tolerance may open new avenues for immune-based interventions with potential impact on the prevention and treatment of macrovascular atherosclerotic disease.

In conclusion, our findings demonstrate that LPS-induced macrophage tolerance represents a protective immunometabolic adaptation that attenuates inflammatory signaling while enhancing HDL-mediated cholesterol efflux. However, this atheroprotective phenotype is selectively disrupted by AGE-RAGE-TLR signaling, highlighting a critical molecular interface through which chronic metabolic stress, represented by AGEs accumulation, may override innate immune tolerance and promote atherosclerotic progression.

## Data Availability

The datasets presented in this study can be found in online repositories. The names of the repository/repositories and accession number(s) can be found below: https://doi.org/10.5281/zenodo.16103900.
